# Prevalence of reflux esophagitis in obese Japanese undergoing bariatric surgery

**DOI:** 10.1002/jgh3.12293

**Published:** 2019-12-17

**Authors:** Kodai Takahashi, Yosuke Seki, Kazunori Kasama, Manabu Amiki, Satoshi Baba, Masayoshi Ito, Tatsuro Tanaka, Eiji Kanehira

**Affiliations:** ^1^ Department of Surgery Medical Topia Soka Saitama Japan; ^2^ Weight Loss and Metabolic Surgery Center Yotsuya Medical Cube Tokyo Japan; ^3^ Department of Gastroenterology Yotsuya Medical Cube Tokyo Japan

**Keywords:** bariatric surgery, gastroesophageal reflux disease, obesity

## Abstract

**Background:**

Currently, the data on the relationship between obesity and gastroesophageal reflux disease (GERD) in Asian populations are scarce.

**Methods:**

The aim of this study is to investigate the prevalence of reflux esophagitis (RE) among obese Japanese patients in each body mass index (BMI) range group. In addition, we aim to investigate the risk factors for RE in obese Japanese patients. The present retrospective cohort study included 674 obese Japanese patients who underwent bariatric surgery between January 2003 and April 2016. The patients were stratified into five groups based on BMI range.

**Results:**

The mean BMI was 42.7 ± 9.24 kg/m^2^. The prevalence of RE among each of the groups was as follows: Group 1 (BMI 30–34.9) = 20.7%; Group 2 (BMI 35–39.9) = 24.0%; Group 3 (BMI 40–44.9) = 25.2%; Group 4 (BMI 45–49.9) = 26.7%; and Group 5 (BMI ≥50) = 24.8%. Overall, the prevalence of RE was 24.2% in our study. Furthermore, no significant difference in BMI was noted between the RE and non‐RE groups (43.4 ± 9.3 kg/m^2^ and 42.5 ± 10.2 kg/m^2^, respectively; *p* = 0.24). According to the multivariate logistic regression model, gender, *Helicobacter pylori* infection status, GERD‐related symptoms, and hiatal hernia were significantly correlated with RE.

**Conclusion:**

Our study shows that the prevalence of RE in severely obese Japanese patients was significantly higher than the average prevalence of RE in Japan. However, the prevalence of RE did not increase with BMI in our cohort.

## Graphical abstract

Our study is the first to report the prevalence of RE in severely obese Japanese patients. Although it has certain limitations, it can still provide valuable reference data in epidemiology.

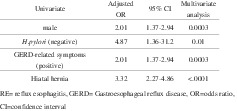

## Introduction

Gastroesophageal reflux disease (GERD) is a disease characterized by a series of troublesome symptoms with complications that arise from the reflux of gastric contents into the esophagus.^1^ Earlier reports have shown that the prevalence of GERD has been estimated to be between 10 and 20% in the United States and Europe, with a lower frequency in Asia.^2^ A recent systematic review estimated the prevalence of GERD in the United States at 18.1–27.8%.^3^ El‐Serag reported that its incidence had significantly increased in the last two decades in North America and Europe but not in Asia.^4^ On the contrary, Fujiwara reported that the prevalence of GERD is increasing in Japan, as well as in the West.^5^

Obesity is a known risk factor for GERD or reflux esophagitis (RE). In earlier studies, several pathophysiological mechanisms linking the two conditions were reported.^6^, ^7^ Several studies have demonstrated a higher prevalence of GERD in obese individuals compared with the nonobese population.^8^, ^9^ Moreover, numerous studies from the United States have shown that increased levels of obesity have been associated with a higher likelihood of GERD.^1^, ^10^, ^11^ The same relationship between obesity and GERD has been observed in Europe.^12^, ^13^, ^14^, ^15^ However, data regarding the relationship between obesity and GERD in Asia have been scarce. One plausible reason for this is the relatively smaller population of severely obese patients among Asian populations. Kang et al. studied 2457 participants who underwent upper gastrointestinal (GI) endoscopy in Korea.^16^ The prevalence rates of RE were 5.6, 8.1, and 15.5% for the body mass index (BMI) groups of <25, 25–30, and > 30 kg/m^2^, respectively. Consequently, it appears that a higher BMI is proportional to the occurrence of RE. Currently, there is insufficient data regarding the relationship between obesity and GERD or RE in other Asian countries. In particular, such a relationship in severely obese Japanese patients has not been reported despite the growing severely obese population in Japan.^17^ This study demonstrates that GERD or RE is prevalent in many severely obese Japanese individuals, which can serve as valuable reference data in epidemiologic studies.

Here, we aim to investigate the prevalence of RE in obese Japanese patients who have undergone bariatric surgery. To the best of our knowledge, this is the first study on this topic.

## Methods

### *Patients*

This retrospective cohort study included 674 consecutive obese Japanese patients (295 females and 379 males) who underwent bariatric surgery between January 2003 and April 2016 at Yotsuya Medical Cube, Tokyo, Japan. All patients provided written informed consent. The inclusion criteria for laparoscopic bariatric surgery were based on the Society of American Gastrointestinal and Endoscopic Surgeons (SAGES) Guidelines for Clinical Application of Laparoscopic Bariatric Surgery (medically uncontrolled, with ages between 18 and 65 years, and a BMI greater than 30 kg/m^2^ with obesity‐related comorbidities), which was approved by the Institutional Review Board. Each patient was preoperatively screened and evaluated by our multidisciplinary team.^18^ The following parameters were evaluated prior to surgery: patient characteristics (gender, age, height, weight, and BMI), visceral fat area, visceral/subcutaneous fat ratio, usage of proton pump inhibitor (PPI) treatment, status of *Helicobacter pylori* (*H. pylori*) infection, GERD‐related symptoms, Barrett's esophagus (BE), and hiatal hernia. Abdominal computed tomography scans were routinely performed to assess visceral/subcutaneous fat measured on one cross‐sectional scan obtained at the level of the umbilicus.

In our cohort, all the patients underwent gastroscopy before bariatric surgery. Regarding the status of *H. pylori* infection, patients with a history of *H. pylori* infection that had already been eradicated were also categorized into the *H. pylori*‐negative group in this study. Prior to the upper GI endoscopy, the participants were interviewed regarding their main complaints and symptoms (chest pain, dyspepsia, and dysmotility). The severity of RE observed on gastroscopy was graded from A to D and was based on the Los Angeles classification system.^19^ On upper GI endoscopy, a hiatal hernia was considered if the diaphragmatic indentation was >2 cm distal to the *Z*‐line and proximal margins of the gastric mucosal folds, which were observed with considerable air insufflation during inspiration. The distance was measured using the centimeter markings during the upper GI endoscopy.^20^, ^21^

The patients were divided into five groups according to their BMI: Group 1 (30–34.9 kg/m^2^), Group 2 (35–39.9 kg/m^2^), Group 3 (BMI 40–44.9 kg/m^2^), Group 4 (BMI 45–49.9 kg/m^2^), and Group 5 (≥50 kg/m^2^). Then, the prevalence of RE in each group was determined. The associations between the visceral fat ratio and BMI were analyzed using Spearman's correlation coefficient. In addition, it was the researcher's aim to investigate the risk factors for RE that were associated with obese Japanese patients.

The present study was approved by the Institutional Review Board to ensure the protection of patient privacy and confidentiality, and it was performed in accordance with the ethical standards of the World Medical Association's Declaration of Helsinki.

### *Statistical analysis*

Statistical analysis was performed using JMP® 11 (SAS Institute Inc., Cary, NC, USA). The results were expressed as the mean ± standard deviation and percentage. Moreover, the grouped data were expressed as the median (range), and nonparametric methods were used. A univariate analysis was performed using the chi‐square test or Fisher's exact test to identify the associations between variables. Furthermore, Cox proportional hazards regression was performed to analyze gender, the status of *H. pylori* infection, GERD‐related symptoms, and hiatal hernia. A probability (*P*) value <0.05 was considered statistically significant. A correlation analysis was also performed using Spearman's correlation coefficient. The study by Guilford (1956) was used to interpret and compare the correlation coefficients. The correlation coefficients he described are discussed below. Here, <0.20: “slight almost negligible relationships”; 0.20–0.40: “low correlation”; 0.40–0.70: “moderate correlation”; 0.70–0.90: “high correlation, marked relationship”; and > 0.90: “very high correlation, very dependable relationship.”

## Results

Regarding patient characteristics, the mean BMI, body weight, and age were 42.7 ± 9.24 kg/m^2^, 117.7 ± 29.4 kg, and 41.2 ± 10.3 years, respectively. On examination, *H. pylori* infection was found in 29 patients (4.3%). The mean visceral fat ratio was 0.39 ± 0.19. GERD‐related symptoms were also noted in 41% of patients. Among the 674 patients, Grades A, B, C, and D were present in 114 cases (16.9%), 37 cases (5.5%), 11 cases (1.5%), and 1 case (0.2%), respectively. In all, the prevalence of RE was 24.2% in our study. Approximately 40% of patients who underwent surgery at our institution had hiatal hernia, and 1.6% had BE. Prior to surgery, 8.9% of patients had already taken PPI medication. Other patient characteristics are shown in Table 1. The prevalence rates of RE in all the groups were as follows: Group 1, 20.7% @@@(*n* = 27/130); Group 2, 24.0% (*n* = 43/179); Group 3, 25.2% (*n* = 35/139); Group 4, 26.7% (*n* = 27/101); and Group 5, 24.8% (*n* = 31/125) (Table 2).

**Table 1:** Patients characteristics

Variables	*n* = 674
Gender, *n* (%)	
Male	379 (56.3)
Female	295 (43.7)
Age (years), mean ± SD	41.2 ± 10.3
BMI	42.7 ± 9.24
*Helicobacter pylori*, *n* (%)	
Positive	29 (4.3)
Negative	645 (95.7)
Visceral/subcutaneous fat ratio, mean ± SD	0.39 ± 0.19
Visceral fat area (cm^2^), mean ± SD	189.40 ± 141.50
GERD‐related symptoms, *n* (%)	
Positive	276 (41.0)
Negative	398 (59.0)
LA classification, *n* (%)	
N	316 (46.9)
M	195 (28.9)
A	114 (16.9)
B	37 (5.5)
C	11 (1.5)
D	1 (0.2)
Barret esophagus, *n* (%)	
Positive	11 (1.6)
Negative	663 (98.4)
Hiatal hernia	
Positive	268 (39.8)
Negative	406 (60.2)
Medication (PPI), *n* (%)	
Positive	60 (8.9)
Negative	614 (91.1)

BMI, body mass index; GERD, gastroesophageal reflux disease; LA, Los Angeles; PPI, proton pump inhibitor; RE, reflux esophagitis; SD, standard deviation.

**Table 2:** The prevalence of RE in each group

BMI (kg/m^2^)	30<	35<	40<	45<	50<
Age (years), mean ± SD	45.5 ± 9.3	41.1 ± 11.4	40.5 ± 10.4	40.2 ± 9.9	38.1 ± 8.3
Number of patients	27/130	43/179	35/139	27/101	31/125
Ratio of patients (%)	20.7	24	25.2	26.7	24.8

BMI, body mass index; RE, reflux esophagitis; SD, standard deviation.

**Figure 1:**
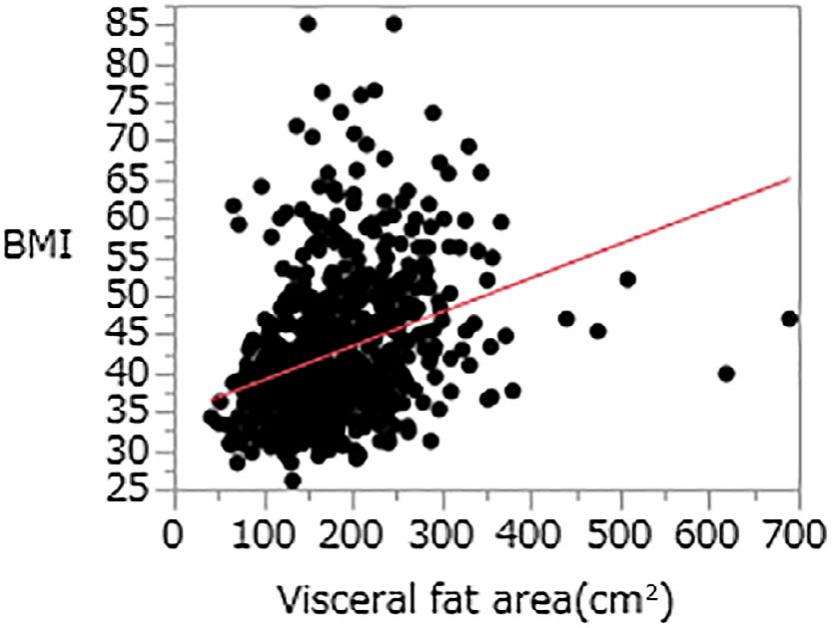
The associations between the visceral fat area and body mass index (BMI) (Spearman's correlation coefficient). No significant correlation between the visceral fat area and BMI was noted (0.39 ± 0.19 and 42.7 ± 9.23, respectively; *P* < 0.0001).

In the univariate analysis, no significant difference in BMI was noted between the RE and non‐RE groups (43.4 ± 9.3 and 42.5 ± 10.2 kg/m^2^, respectively; *P* = 0.24) (Table 3). Furthermore, no significant correlation was observed between the visceral fat ratio and BMI (0.39 ± 0.19 and 42.7 ± 9.23, respectively; *P* < 0.0001; Spearman's correlation coefficient) (Fig. 1). No significant differences were noted in age, visceral/subcutaneous fat ratio, BE, and usage of PPI therapy between the RE and non‐RE groups. Male gender was more prevalent in the RE group (*P* < 0.0001). The proportion of patients who experienced GERD‐related symptoms and hiatal hernia was significantly higher in the RE group compared with the non‐RE group (*P* < 0.0001 and *P* < 0.0001, respectively, for the RE group and non‐RE group). On the contrary, the proportion of patients with *H. pylori* infection was significantly lower in the RE group (*P* < 0.02) (Table 4). According to the multivariate logistic regression model, gender, status of *H. pylori* infection, GERD‐related symptoms, and hiatal hernia were significantly correlated with RE (Table 5).

**Table 3:** The associations between RE and BMI

LA classification	M, N	A, B, C, D	Univariate analysis (*P*)
Number of patients	163	511	0.24
BMI (kg/m^2^)	43.4 ± 9.3	42.5 ± 10.2	

BMI, body mass index; RE, reflux esophagitis.

**Table 4:** The risk factors for RE in obese Japanese patients

Variables	RE (+)	RE (−)	Univariate analysis (*P*)
Gender, *n* (%)			<0.0001
Male	95 (14.1)	200 (29.7)	
Female	68 (10.1)	311 (46.1)	
Age (years), mean ± SD	41.8 ± 9.5	40.9 ± 10.5	0.22
*Helicobacter pylori*, *n* (%)			<0.02
Positive	2 (0.3)	27 (4.0)	
Negative	161 (23.9)	484 (71.8)	
Visceral/subcutaneous fat ratio, mean ± SD	0.4 ± 0.17	0.38 ± 0.19	0.11
Visceral fat area (cm^2^), mean ± SD	195.07 ± 74.35	187.59 ± 157.01	0.55
GERD‐related symptoms, *n* (%)			<0.0001
Positive	88 (13.1)	188 (27.9)	
Negative	75 (11.1)	323 (47.9)	
Barret esophagus, *n* (%)			0.34
Positive	4 (0.6)	7 (1.0)	
Negative	159 (23.6)	504 (74.8)	
Hiatal hernia, *n* (%)			<0.0001
Positive	104 (15.4)	164 (24.3)	
Negative	59 (8.8)	347 (51.5)	
Medication (PPI), *n* (%)			0.08
Positive	9 (1.3)	51 (7.6)	
Negative	154 (22.9)	460 (68.2)	

GERD, gastroesophageal reflux disease; PPI, proton pump inhibitor; RE, reflux esophagitis; SD, standard deviation.

**Table 5:** Multivariate logistic regression model associated with RE

Univariate	Adjusted OR	95% CI	Multivariate analysis (*p‐value*)
Male	2.01	1.37–2.94	0.0003
*Helicobacter pylori* (negative)	4.87	1.36–31.2	0.01
GERD‐related symptoms (positive)	2.01	1.37–2.94	0.0003
Hiatal hernia	3.32	2.27–4.86	<0.0001

CI, confidence interval; GERD, gastroesophageal reflux disease; OR, odds ratio; RE, reflux esophagitis.

## Discussion

The aim of this study is to evaluate the prevalence of RE in severely obese Japanese patients stratified according different ranges of BMI. In this study, only severely obese Japanese patients with a BMI greater than 30 were included. To date, no data of this scale with this demographic are available in literature reviews. We believe that the information gained from this study could demonstrate the relationship between obesity and RE among Asian populations, particularly among obese Japanese.

Obesity has increased globally, and more than 30% of adults are considered to have a BMI value exceeding 25.0 kg/m^2^.^22^ Although the obesity rate in Japan is the lowest among the Organization for Economic Co‐operation and Development‐affiliated countries, this rate has increased among men and the elderly. In a national health and nutrition survey conducted in 2017, 30.7% of adult males and 21.9% of adult females in Japan had a BMI exceeding 25 kg/m^2^. Theoretically, the prevalence of GERD or RE will eventually increase with the increase in obesity rates. A recent study showed that the prevalence rates of GERD were 6.2%, 2.5–4.8%, and 3.5% in China, Hong Kong, and Korea, respectively.^23^, ^24^, ^25^, ^26^, ^27^ Based on earlier studies on Asian populations, the prevalence of GERD or RE in Asia remains lower than that reported in the West.^2^, ^3^, ^8^, ^9^, ^28^, ^29^ Although the percentage of obese Asians may be low, epidemiological studies regarding GERD or RE complicate the comparison because of the differences in its prevalence caused by environmental factors and genetic variations in the populations in Asia and the West.^30^ Accordingly, investigations on the prevalence of GERD or RE in Asia and in severe obese individuals would be extremely useful as only a few studies on this topic have been reported.^16^, ^26^, ^31^ A recent Japanese epidemiological study investigated the prevalence of GERD between normal‐weight and overweight or obese patients.^32^ This study showed that the prevalence rates of RE were significantly higher in overweight (25.8%) (odds ratio [OR], 2.27; 95% confidence interval [CI], 1.82–2.82) and obese (35.9%) (OR, 3.65; 95% CI, 2.40–5.57) patients compared with normal‐weight patients. Although related studies about the prevalence of GERD or RE based on differences in body weight have been previously reported,^32^ this is the first study that compares the prevalence of RE in severely obese Japanese patients according to their BMI.

In general, the prevalence of RE was 6.1–13.7% in Japan.^5^, ^33^, ^34^, ^35^, ^36^ The present study shows that the prevalence of RE in severely obese Japanese patients was significantly higher than the average prevalence of RE in Japan. Obesity may increase the risk of RE, which can be attributed to mechanical factors.^6^, ^7^, ^37^, ^38^, ^39^ From a physiologic standpoint, abdominal obesity increases intra‐abdominal pressure.^40^, ^41^ If the intra‐abdominal pressure increases, both gastric and esophageal pressures also increase, and the lower esophageal sphincter (LES) may be displaced in a cephalad fashion.^42^ Therefore, an increased intra‐abdominal pressure in obese individuals may cause RE. The previous statement is supported by Wilson's observation: a higher prevalence of hiatal hernia in obese patients may contribute to an increased rate of RE in the same population.^43^ Fisher and Mercer, respectively, noted that the gastroesophageal pressure gradient and the frequency of transient LES relaxation were significantly higher in obese patients.^6^, ^44^ Theoretically, with an increase in BMI, the abdominal pressure was predicted to be high, thereby increasing the prevalence of RE. However, the prevalence of RE did not increase with BMI in our cohort.

The possible causes of the increase in BMI are not necessarily an increase in visceral fat but an increase in subcutaneous fat. In this study, no correlation between the BMI and visceral fat ratio was noted. Therefore, the prevalence of RE in obese patients was higher than that in normal‐weight patients, but RE presumably did not increase with increasing BMI among severely obese patients.

Several reports comparing normal‐weight and obese patients have identified a strong association between an increased BMI and GERD‐related symptoms, although all these differ in their observations.^43^, ^45^, ^46^ Furthermore, the prevalence of GERD in obese patients was higher than that in normal‐weight patients in the West, but it did not necessarily increase with the BMI.^8^, ^47^

An earlier Japanese study demonstrated that the proportions of participants with GERD‐related symptoms were 23.3, 26.7, and 50% for the BMI groups of <25, 25–30, and > 30 kg/m^2^, respectively, and that the corresponding prevalence rates of RE were 12.5, 29.8, and 26.9%.^32^ In a recent report comparing the prevalence of RE in Japanese normal‐weight patients compared with obese patients, obesity was significantly different when considering the increased prevalence of RE, but it was not significantly different in GERD.^32^ In addition, a comprehensive assessment of the data from earlier studies suggests that the prevalence of GERD or RE can be significantly increased in obese patients compared with normal‐weight patients.^43^, ^45^, ^46^ However, the data obtained in this study suggest that a sole relationship between obesity grade and prevalence of RE among obese patients cannot be established.

Recently, esophageal hiatal hernia, *H. pylori* infection, and gender have been reported as risk factors for RE in Europe, United States, and Asia.^30^, ^48^, ^49^, ^50^, ^51^ According to this study, similar results were obtained for severely obese Japanese patients.

This study has some limitations that are inherent to observational studies that should be addressed. First, Grade M of GERD is a classification unique to Japan; its comparison with grades in other foreign studies is difficult. Bias cannot be totally excluded when the diagnosis of Grade M is considered, especially in the presence of an enforcer. As multiple enforcers were involved in this examination, the introduction of a bias is highly possible. Second, because this study examined patients who underwent bariatric surgery, the population was isolated. Inevitably, this study was focused on a group of young patients with several comorbid obesity‐related diseases, and therefore, such a group may not, in general, correctly reflect severely obese Japanese in the general population. Nonetheless, a relatively large number of cases was involved, and endoscopic examinations were performed in all the cases as a preoperative evaluation step. This is a retrospective study, but it may be highly reliable because it is consecutive and has no selective bias. Finally, the present study evaluated the prevalence of RE and did not accurately evaluate nonerosive reflux disease (NERD). Technically, endoscopy and 24‐h pH monitoring impedance testing are required to properly diagnose NERD. However, pH monitoring and impedance testing are practically difficult to conduct in all severely obese patients who have GERD‐related symptoms but who do not primarily intend to undergo treatment for RE. In addition, 8.9% patients had already undergone PPI therapy before surgery, which may have caused bias.

## Conclusion

Our study is the first to report the prevalence of RE in severely obese Japanese patients. We show that the prevalence of RE in severely obese Japanese patients was significantly higher than the average prevalence of RE in Japan. However, no significant difference was observed in BMI between the RE and non‐RE groups. Although our study has certain limitations, these findings can still provide valuable reference data in the epidemiology field. In the future, a well‐designed, large‐sample, multicenter study is required to confirm these findings.
